# Anemia and the onset of gout in a population-based cohort of adults: Atherosclerosis Risk in Communities study

**DOI:** 10.1186/ar4026

**Published:** 2012-08-20

**Authors:** Mara A McAdams-DeMarco, Janet W Maynard, Josef Coresh, Alan N Baer

**Affiliations:** 1 Department of Epidemiology, Johns Hopkins University Bloomberg School of Public Health, 615 N. Wolfe St, Baltimore, MD 21205, USA; 2 Department of Surgery, Johns Hopkins University School of Medicine, 720 Rutland Ave, Baltimore, MD 21205, USA; 3 Division of Rheumatology, Johns Hopkins University School of Medicine, 5200 Eastern Avenue, Mason F. Lord Building, Center Tower, Suite 4100; Baltimore, MD 21224, USA

## Abstract

**Introduction:**

There is a growing prevalence of gout in the US and worldwide. Gout is a recognized risk factor for cardiovascular disease (CVD). It is unclear whether other risk factors for CVD are also associated with increased risk of gout. Anemia is one such CVD risk factor. No studies have evaluated the relationship between anemia and gout. We tested whether anemia was associated with incident gout independent of comorbid conditions in Atherosclerosis Risk in the Communities.

**Methods:**

This population-based cohort recruited 15,792 individuals in 1987 to 1989 from four US communities and contained nine years of follow-up. Anemia was defined as hemoglobin <13.5 g/dL for men and <12 g/dL for women. Using a Cox Proportional Hazards model, we estimated the hazard ratio (HR) and confidence intervals (CI) of incident gout by baseline anemia, adjusted for confounders (sex, race, estimated glomerular filtration rate, body mass index and alcohol intake) and clinical factors (coronary heart disease, congestive heart failure, diabetes, hypertension, diuretic use and serum urate level).

**Results:**

Among the 10,791 participants, 10% had anemia at baseline. There were 271 cases of incident gout. Patients with anemia had a two-fold increased risk of developing gout over nine years (HR = 2.01, 95% CI: 1.46, 2.76). Anemia was associated with incident gout independent of known gout risk factors, confounders and clinical risk factors (HR = 1.73, 95% CI: 1.24, 2.41). This association persisted after additionally adjusting for serum urate level (HR = 1.83, 95% CI: 1.30, 2.57).

**Conclusion:**

We identified anemia as a novel risk factor for gout. Anemia was associated with an approximately two-fold increased risk of gout-independent kidney function and serum urate. These findings suggest that anemia is a risk factor for gout on par with other chronic conditions such as obesity and diabetes. The biological mechanism linking anemia to gout remains unclear.

## Introduction

The prevalence of gout is increasing in the United States; in 2005, the estimated prevalence was 3 million cases, which has increased from 2.1 million in 1995 [1]. Serum urate levels are recognized as a key risk factor for the development of gout [2], and those with serum urate levels above 8.0 mg/dL have an incidence rate of gout greater than 25/1,000 person-years [3]. However, in one study, less than 22% of individuals with hyperuricemia developed gout over five years [2], suggesting additional factors influence the progression from hyperuricemia to the development of gout. Current research has emphasized the effect of traditional cardiovascular risk factors on the development of gout, such as obesity, hypertension and dietary factors [4-6]. Additionally, gout is an independent risk factor for myocardial infarction [7], as well as all cause and cardiovascular mortality [8,9].

It is unclear whether other risk factors for cardiovascular disease (CVD) are also associated with increased risk of gout. Anemia is one such risk factor, and is associated with CVD [10], chronic diseases [11,12] and mortality, as well as a decreased quality of life in patients with chronic disease [13-16]. One potential biological pathway linking anemia to gout is oxidative stress; oxidative stress is increased in anemia [17], hyperuricemia is a consequence of increased oxidative stress. Although, anemia is an established risk factor for CVD, no studies have tested whether anemia increases the risk of gout. Additionally, it is unclear whether anemia is related to the development of gout independent of comorbid conditions that are common to both anemia and gout, such as kidney function.

We hypothesized that anemia is associated with an increased risk of developing gout. Further, we postulated that the relationship exists above and beyond the effect of serum urate levels and kidney function. We sought to evaluate the independent association of anemia and gout, after controlling for possible confounders over nine years of follow-up in a longitudinal population-based cohort of middle-aged adults.

## Materials and methods

### Setting and participants

The Atherosclerosis Risk in the Communities study (ARIC) is a prospective population-based cohort study of 15,792 individuals recruited from four US communities (Washington County, Maryland; Forsyth County, North Carolina; Jackson, Mississippi; and suburbs of Minneapolis, Minnesota). The Institutional Review Board of the participating institutions approved the ARIC study protocol and study participants provided written informed consent. Participants aged 45 to 64 years were recruited to the cohort in 1987 to 1989. This cohort was established to study the natural history of atherosclerosis, and the study consisted of one baseline visit (visit 1) between 1987 and 1989 and three follow-up visits (visits 2, 3, and 4) administered three years apart. Details of the study design have been previously published [18].

This analysis was limited to participants who were Caucasian or African American; few participants reported other races (n = 48). We excluded participants who did not report their gout status at visit 4 (n = 4,269) and those with prevalent gout at cohort entry, defined as the self-report of gout onset prior to the baseline visit (n = 419). Participants with missing baseline information on the main covariates of interest were not included (n = 265; sex, race, estimated glomerular filtration rate (eGFR), Body Mass Index (BMI), alcohol intake and hypertension). Participants who were older, had diabetes, lower education and higher serum urate were more likely not to be included in this analysis. However, baseline hypertension, BMI, eGFR, use of an antihypertensive medication (including diuretic use) or sex were associated with being included in this analysis. Therefore, it is unlikely that our results are not generalizable to the ARIC cohort.

There were 10,791 participants included in this study of the association of anemia and gout. This study hypothesis was developed *a priori *and the sole focus of this analysis.

### Exposure: baseline anemia status

At baseline, each participant had their blood drawn and the procedures have been described elsewhere [19]. Hemoglobin was measured using automated hematology analyzers (Coulter S + IV (calibration S-Cal), Beckman Coulter, Inc, Fullerton, CA, USA) at two sites, Coulter S + III and Coulter S + IV (calibration S-Cal) at one site and Technicon H-6000 (calibration Fisher, Technicon Corporation, Tarrytown, NY, USA) at one site. Anemia was defined as baseline hemoglobin less than or equal to 12 g/dL for female participants and less than or equal to 13.5 g/dL for male participants. The cutpoint for women is the same as the World Health Organization (WHO) definition of anemia [20]. However, for men, we chose 13.5 g/L for anemia rather than the WHO cutpoint for men (13.0 g/L) [20] because it approximated the lowest deciles for men (12 g/dL was the lowest decile for women) and was consistent with previous ARIC studies [11].

### Outcome: incident gout

At ARIC visit 4, participants were asked, "Has a doctor **ever **told you that you had gout?" Participants who answered, "Yes," to the gout query then reported the age at gout diagnosis. The outcome of interest was incident gout based on self-reported onset of gout at an age greater than their age at ARIC visit 1. Our previous research suggests that self-report of a physician diagnosis of gout is a reliable (three-year reliability kappa = 0.73) and a sensitive (sensitivity = 84%) measure of gout [21].

### Confounders and characteristics

Other covariates of interest at baseline (1989) included self-reported age (in years), sex (male or female), race (white or African American), education (<12 years, 12 to 16 years and 17 to 21 years) and alcohol intake (grams/week). Additionally, we included clinical factors that were thought to be related to anemia or gout, including diabetes (fasting glucose level ≥126 mg/dL, nonfasting glucose level ≥200 mg/dL, reported physician-diagnosis of diabetes, or history of medication use for diabetes), measured BMI (kg/m^2^), coronary heart disease (CHD) (myocardial infarction from adjudicated visit 1 ECG data, history of myocardial infarction by self-report or history of heart or arterial surgery, report of coronary bypass, balloon angioplasty, angioplasty of coronary arteries), hypertension (self-report of medication to treat hypertension, or a measured blood pressure ≥140/90 mm Hg) or diuretic use in the two weeks prior to the baseline visit. Self-report of a history of heart failure or coronary heart failure (CHF) at visit 4 was also considered. Serum creatinine was estimated using a modified kinetic Jaffé reaction. Glomerular filtration rate (eGFR) was estimated using the CKD-Epi equation [22] and categorized as ≥90, 60 to 90, or <60 ml/min/1.73 m^2^.

Serum urate concentrations were measured with the uricase method at visit 1 in mg/dL. The reliability coefficient of serum urate was 0.91, and the coefficient of variation was 7.2% in a sample of 40 individuals with repeated measures taken at least one week apart [23].

### Statistical methods

The mean and standard deviation (SD), as well as the prevalence of the covariates, were calculated and compared by baseline anemia status using a *t*-test and chi-squared test, respectively.

Using a Cox Proportional Hazards model, the hazard ratio (HR) and 95% confidence interval (CI) of incident gout by baseline anemia was estimated with age as the time scale. The unadjusted survival functions were plotted using Kaplan-Meier plots. Models were adjusted for confounders of the association of anemia and gout, including sex and race, as well as baseline measures of BMI, categorical eGFR and alcohol intake. In a separate model, we additionally adjusted for baseline diabetes, hypertension, CHD, history of CHF and diuretic use. All of these measures were considered confounders of the gout and anemia relationship, based on exploratory data analysis and previous research [5,24,25]. We hypothesized that the presence of uterine fibroids may explain the observed association of anemia and gout in the female population. Thus, in an analysis limited to women, we adjusted for surgical menopause as a proxy because fibroids are the leading cause of hysterectomy. Additionally, we adjusted for baseline serum urate level to test whether the association of anemia and gout was independent of serum urate levels.

We tested whether the association of anemia and incident gout differed by sex, race and eGFR (<60 ml/min/1.73m^2 ^or < 60 ml/min/1.73m^2^). The stratified models were adjusted for the previously listed confounders. Using a Wald test, we assessed whether these factors were effect measure modifiers (statistical interaction) of the association of anemia and incident gout.

As a sensitivity analysis, we restricted the population to those with an eGFR> 30 ml/min/1.73m^2 ^and tested the association of anemia and gout. Additionally, we examined whether the association of anemia and incident gout differed when the model was adjusted for time-varying eGFR, including estimates of GFR from visit 1 and 2. We tested whether socio-economic status factors and other risk factors explained the association between anemia and gout: education, smoking status, fibrinogen (an acute phase reactant), use of non-steroidal anti-inflammatory drugs (NSAIDs), mean corpuscular volume and for women menopausal status and history of surgical menopause.

All analyses were performed in SAS, version 9.1 (SAS Institute, Cary, NC, USA). The cumulative incidence plots were constructed in Stata, version 11 (Stata Corp, College Station, TX, USA).

## Results

### Study population characteristics

**Figure 1:**
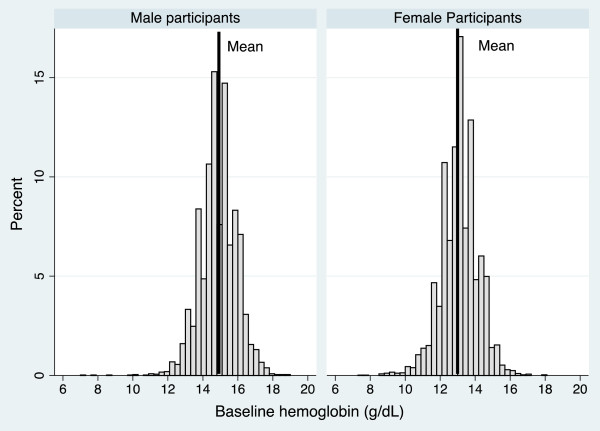
**Distribution of hemoglobin for male and female participants in the ARIC study**. The solid line displays the mean in each group (Males = 14.9 g/dL, SD = 1.05; Females 13.1 g/dL, SD = 1.05).

A total of 10,791 ARIC participants met the study criteria. The study population was 43% male, 21% African American and the mean age at cohort entry was 54 years (SD = 5.70). The mean hemoglobin level was 13.9 g/dL (SD = 1.37); for men 14.9 g/dL (SD = 1.05) and for women 13.1 g/dL (SD = 1.05) (Figure 1). At baseline, 1,084 (10%) participants were classified as having anemia; 66% of the participants with anemia were women.

There were 271 cases of incident gout. Of the 271 gout cases, 39% were female and 38% were African American. The mean age at baseline for the gout cases was 54 (SD = 5.9) and the mean age at gout diagnosis was 59 (SD = 6.4). At baseline, 64% of the women with gout were post-menopausal. The mean serum urate level at baseline was 8.0 mg/dL (SD = 2.0) for those who developed gout during follow-up.

Table 1 lists the study population characteristics and gout risk factors by baseline anemia status. Participants with anemia were more likely to be female and African American race than those without anemia. Those with anemia had a lower alcohol intake, serum urate level and lower educational attainment. Participants with eGFR <60 ml/min/1.73m^2 ^were more likely to have anemia (2.6% vs. 1.9%, *P*-value <0.001 for trend).

**Table 1:** Baseline gout risk factors by baseline anemia status in the ARIC study.

	*No anemia *	*Anemia *
**Baseline gout risk factors**	***N = 9,707***	***N = 1,084***

**Male sex**, n (%)	4,315 (44)	364 (34)**
**Mean age**, years (SD)	53.9 (56)	53.1 (6)**
**African American race**, n (%)	1,738 (18)	549 (51)**
**Hypertension**, n (%)	2,878 (30)	344 (32)
**Diuretic use**, n (%)	1,478 (15)	160 (15)
**Diabetes**, n (%)	834 (9)	94 (9)
**Coronary heart disease**, n (%)	304 (3)	37 (3)
**Congestive heart failure**, n (%)	209 (2)	30 (3)
**Mean BMI**, kg/m^2^, (SD)	27.4 (5)	27.5 (6)
**Mean ethanol intake**, grams/week, (SD)	41.9 (90)	25.9 (62)**
**eGFR**, n (%)		
<60 ml/min/1.73m^2^	186 (2)	28 (3)
60 to 90 ml/min/1.73m^2^	4,140 (43)	337 (31)
≥90 ml/min/1.73m^2^	5,381 (55)	719 (66)**
**Education level**, n (%)		
<12 years	1,755 (18)	274 (25)
12 to 16 years	4,162 (43)	418 (39)
17 to 21 years	3,781 (39)	388 (36)**
**Mean serum uric acid**, mg/dL (SD)	5.9 (2)	5.7 (2)**
**Incident gout**, n (%)	224 (2.3)	47 (4.3)**

* *P*-value <0.05 ; ** *P*-value <0.001 comparing anemia to no anemia. Anemia was defined as hemoglobin <13.5 g/dL for men and <12 g/dL for women.

### Association of anemia and incident gout

**Figure 2:**
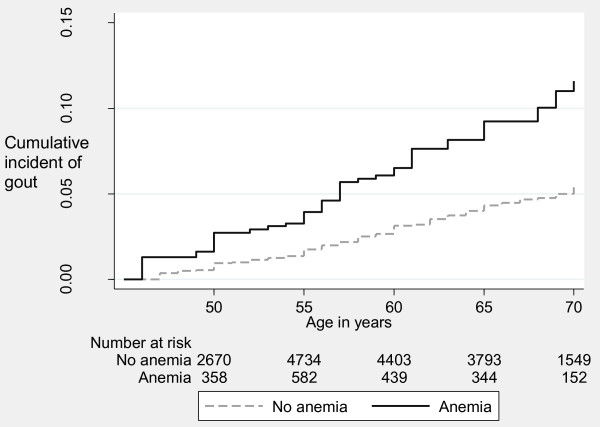
**Cumulative incidence of incident gout by baseline anemia status in the ARIC study**. Log-rank *P*-value <0.0001.

Incident gout occurred more frequently in participants with anemia at baseline compared with those without anemia (nine-year cumulative incidence: 4.3% vs. 2.3%, *P*-value <0.001), Table 1. Figure 2 presents the Kaplan-Meier cumulative incidence function of gout by baseline anemia status, suggesting that by age 70, the cumulative incidence of gout was 4.4% in those without anemia and 10.2% in those with anemia. Table 2 lists the unadjusted and adjusted HR of incident gout by baseline anemia status. Participants with anemia were twice as likely to develop gout than participants without anemia (HR = 2.01, 95% CI: 1.46, 2.76). Baseline anemia was associated with a 1.7-fold (95% CI: 1.24, 2.41) increase in the hazard of incident gout after adjusting for clinical risk factors for gout and confounders of the anemia and gout association. Even after adjustment for serum urate level, anemia was associated with incident gout (HR = 1.83, 95% CI: 1.30, 2.57).

**Table 2:** Hazard ratio (HR) of incident gout by baseline anemia in the ARIC study.

	*No anemia*	*Anemia*
*Model*	*HR (95% CI)*	*HR (95% CI)*

Unadjusted	Reference	2.01 (1.46, 2.76)**
Sex and race adjusted	Reference	1.52 (1.10, 2.12)*
Sex, race and eGFR adjusted	Reference	1.54 (1.11, 2.14)*
Adjusted for confounders^1^	Reference	1.64 (1.18, 2.28)*
Additionally adjusted for clinical factors^2^	Reference	1.73 (1.24, 2.41)*
Additionally adjusted for serum urate	Reference	1.83 (1.30, 2.57)**

^1 ^Confounders: Sex, race, categorical eGFR (CKD-EPI), body mass index (kg/m^2^) and alcohol intake. ^2 ^Clinical factors: Baseline hypertension, diuretic use, coronary heart disease, and congestive heart failure. * *P*-value <0.05. ** *P*-value <0.001. Note: Age was used as the time-scale. Anemia was defined as hemoglobin <13.5 g/dL for men and <12 g/dL for women.

### Effect measure modification of the anemia and gout association

There was no evidence of effect measure modification by race (*P*-value = 0.83) or kidney function (*P*-value = 0.29). However, there was limited support for an interaction by sex (*P*-value = 0.06). Among female participants, the HR of incident gout by baseline anemia status was 2.38 (95% CI: 1.47, 3.84) compared to a HR of 1.43 (95% CI: 0.88, 2.30) for male participants.

### Sensitivity analyses

In an analysis restricted to those who had an eGFR greater than 30 ml/min/1.73 m^2^, the association of anemia and incident gout was similar to the main analysis (Adjusted HR: 1.80; 95% CI: 1.29, 2.52). Adding visit 2 eGFR to the model as a time-varying confounder did not change the results (results not shown). Although we did not have a direct measure of socioeconomic status, the results did not differ when education and baseline smoking status were added to the multivariate model (Adjusted HR: 1.73, 95% CI: 1.23, 2.42). Adjusting for baseline NSAIDs use did not alter the association between anemia and gout (Adjusted HR: 1.74, 95% CI: 1.25, 2.43). Additionally, in the models restricted to women, the results did not change when we additionally adjusted for menopause (Adjusted HR: 2.02, 95% CI: 1.24, 3.28). When we adjusted for baseline history of surgical menopause, as a proxy for fibroids [26], the association of anemia and gout did not change (Adjusted HR: 2.43, 95% CI: 1.51, 3.92). Finally, adjusting for mean corpuscular volume, as a proxy for hemoglobinopathy, did not alter the association of anemia and gout (Adjusted HR: 1.83, 95% CI: 1.30, 2.58).

## Discussion

This study is, to our knowledge, the first to identify anemia as a risk factor for gout over nine years of follow-up in a large population-based study of middle-aged adults. Even after adjustment for kidney function, comorbid conditions, and education and baseline smoking status, anemia remained an independent predictor of incident gout. Unlike traditional risk factors, anemia was associated with gout, even after adjustment for urate levels, suggesting an independent pathway. No differences in the association between anemia and gout were observed by race, or baseline kidney function. However, there is slight evidence of a difference in the association between anemia and gout between men and women, with a stronger association observed for women.

Although, at this time there is no clear biological link between anemia and gout, there are potential explanations for this observed association. Anemia is an independent risk factor for the development of adverse cardiovascular outcomes, both short-term [27,28] and long-term [10,29-32]. Previous studies have identified anemia as a risk factor for CVD and independent of chronic kidney disease (CKD). For example, anemia was associated with an adjusted HR of 1.41 (95% CI: 1.01, 1.95) for incident CVD [10], and in a population of patients with normal kidney function, anemia was associated with first cardiovascular-specific hospitalizations (HR = 2.49, 95% CI: 1.99, 3.12) [33], supporting the role of anemia as an independent risk factor above and beyond the effects of kidney function [14]. Renal tubular dysfunction can occur in the absence of renal insufficiency (as measured by the creatinine clearance), so alterations in renal urate excretion could occur if the anemia was related to lead poisoning, chronic aspirin use, sickle cell and early CKD (particularly polycystic kidney disease).

Additionally, anemia increases cardiovascular mortality in specific disease states, such as CKD, both in dialysis patients [34] and intermediate CKD stages [11,35]; as well as CHF [34], diabetes [36] and myelodysplastic syndrome [37]. There has been tremendous interest in anemia as a risk factor for CVD because it is potentially modifiable with iron therapy or erythropoietin [38].

Oxidative stress is increased in anemia [17], perhaps particularly in iron-deficiency anemia [39] where iron-deficiency affects catalase activity. The increased oxidative stress could have long-term consequences. One such consequence is induction of hyperuricemia due to increased xanthine oxidase activity, increased cell death/turnover, alteration of cellular macromolecules making them more amenable to urate crystallization (a mechanism akin to the role of oxidized lipoproteins contributing to atherosclerotic plaques) or impairment in renal urate excretion (increased lactate production, increased urate reabsorption because of hypoxic signal to kidney) [17].

Serum urate levels are thought to be positively correlated with iron levels [40-42]. Previous evidence supports a role for iron deficiency in the pathogenesis of gout; when iron is added to media containing urate crystals there is a stimulated oxidative stress with subsequent complement and neutrophil activation. Conversely, the removal of iron inhibits these responses and maintenance of near iron deficiency diminished gouty attacks in patients with gout [43]. However, these results are not in conflict with the reported findings. Anemia may be associated with other chronic disease not captured in ARIC or low B12 folate levels. Furthermore, patients with anemia may also be taking iron supplements, which increase iron levels and thus leads to increased serum urate level and gout risk.

Both gout and anemia are associated with CKD [44,45] and anemia may be a marker for the duration and severity of kidney disease. Thus, anemia could be a proxy for CKD and the association of anemia and incident gout could have been confounded by CKD. However, we found that anemia remained statistically associated with the development of gout after adjustment for kidney function and in participants without renal impairment. Therefore, anemia may be a marker of gout independent of kidney function, as has been demonstrated with CVD [14].

Anemia could also be a marker of chronic conditions and not an independent risk factor for gout. Previous studies have identified anemia as predictor of mortality and morbidity [11,13-15,33]. In our study, a baseline history of chronic conditions was not associated with anemia although in the same cohort, anemia was associated with incident CVD [10]. Additionally, the presence of chronic diseases did not explain the association of anemia and gout. However, another feature of chronic diseases, such as inflammation, may be the link between anemia and gout. Inflammation occurs in patients with anemia [46,47] and urate crystals trigger inflammation during a gout attack [48]. Additionally, inflammation has been found to occur more often in older anemic patients than younger patients [47], which may explain why this association was first observed in a middle-aged adult population. Although, our results were similar when fibrinogen, an acute phase reactant, was included in the multivariate model, we cannot rule out the possibility that inflammation or another factor confounds the association of anemia and gout.

Additionally, different female-specific health factors may be driving the association of anemia and incident gout. Anemia was much more common in women and traditional gout risk factors, such as BMI, alcohol intake and diuretic use, are stronger predictors of gout in men compared to women [3]. Our results provide slight evidence that the association between anemia and gout may exist for women but not men. African-American women typically have a higher prevalence of iron-deficiency anemia due to the bleeding associated with fibroids [26]. Fibroids are also associated with increased BMI and hypertension, factors which may affect the development of gout, and this may be one explanation for the observed results [26]. However, in the models restricted to women, when we adjusted for history of surgical menopause, the best proxy available in these data, anemia remained associated with the development of gout.

Finally, anemia may be a cause of gout. Although it is unclear whether the observed association of anemia and gout is causal, anemia is thought to be more than just a bystander on the biological pathway between anemia and chronic conditions [16]. We did not identify possible biological explanations of the observed association of anemia and gout; although, it is possible that anemia causes gout. However, this study was not designed to establish causality.

### Strengths and limitations

To our knowledge, this is the first study to identify anemia as a risk factor for incident gout. This is one of the largest biracial studies of gout, which included both men and women with gout. Anemia was established prior to the onset of gout, thus supporting temporality. Additionally, we were able to control for kidney function at baseline and other important risk factors for both gout and anemia. For example, we adjusted for race, hypertension and kidney function, which are known common risk factors for gout and anemia [5,46,49]. Finally, our measure of socioeconomic status and education did not alter our results. Additionally, ARIC is one of the few studies that has measures of serum urate and collected data on incident gout during follow-up. This allowed us to test whether the association of anemia and gout remained after adjustment for serum urate levels.

As with any epidemiology study there are limitations to our study. The main limitation of our study was that gout was self-reported by participants at visit 4 and incidence of gout was based on reported age at onset. However, our previous work has suggested that self-reported gout and age of onset is both sensitive and reliable [21]. Participants had to survive until visit 4 and be healthy enough to attend the clinical visit to be included in this study. This may induce bias if those who attended visit 4 were different from the study population with respect to the association between anemia and gout. However, if anemia is non-differentially related to mortality, then this would bias our results towards the null, suggesting that our results are an underestimate of the true association. The etiology of participants' anemia could not be determined in this study and we were unable to establish whether participants had a hemoglobinopathy. Additionally, it is not known whether participants were taking iron supplements. Finally, the hemoglobin cutpoints for anemia were relatively high as severe anemia, hemoglobin <11 g/dL, was rare in this population as it would be in the general population. However, previous studies of anemia have used hemoglobin levels and not established the etiology [10,11]. We cannot be assured that we have eliminated residual confounding, although we have adjusted for the main confounders as was previously noted.

## Conclusions

Our population-based, longitudinal study is the first to identify anemia as a novel risk factor for gout in middle-aged men and women of both races. Future studies should not only confirm the risk of gout associated with anemia but also further elucidate the biological pathways of anemia and gout by measuring epoetin and the use of iron supplements. In particular, the role of anemia on the onset of gout in women should be explored in greater detail, especially with a prospective ascertainment of fibroids.

## Abbreviations

ARIC: Atherosclerosis Risk in the Communities study; BMI: body mass index; CHD; coronary heart disease; CHF: coronary heart failure; CKD: chronic kidney disease; CVD: cardiovascular disease; eGFR: glomerular filtration rate; SD: standard deviation; WHO: World Health Organization

## Competing interests

The authors declare that they have no competing interests.

## Authors' contributions

MMD made substantial contributions to conception and design, acquisition of data, analysis and interpretation of data, and was involved in drafting the manuscript and revising it critically for important intellectual content. JM made substantial contributions to conception and design, interpretation of data, and was involved in revising the manuscript critically for important intellectual content. JC made substantial contributions to conception and design, acquisition of data, analysis and interpretation of data, and was involved in drafting the manuscript or revising it critically for important intellectual content. AB made substantial contributions to conception and design, acquisition of data, analysis and interpretation of data, and was involved in drafting the manuscript and revising it critically for important intellectual content. All authors gave final approval of the version to be published.
